# Intimate partner violence and its association with maternal depressive symptoms 6–8 months after childbirth in rural Bangladesh

**DOI:** 10.3402/gha.v7.24725

**Published:** 2014-09-12

**Authors:** Zarina N. Kabir, Hashima-E Nasreen, Maigun Edhborg

**Affiliations:** 1 Division of Nursing, Department of Neurobiology, Care Sciences and Society, Karolinska Institute, Stockholm, Sweden; 2 Department of Community Medicine, Kulliyyah of Medicine, International Islamic University Malaysia, Kuala Lumpur, Malaysia; 3 Formerly Research and Evaluation Division, BRAC, Dhaka, Bangladesh

**Keywords:** intimate partner violence, mental health, South Asia, Bangladesh, maternal depression

## Abstract

**Background:**

The prevalence of intimate partner violence (IPV), a gross violation of human rights, ranges widely across the world with higher prevalence reported in low- and middle-income countries. Evidence related mainly to physical health shows that IPV has both direct and indirect impacts on women's health. Little is known about the impact of IPV on the mental health of women, particularly after childbirth.

**Objective:**

To describe the prevalence of IPV experienced by women 6–8 months after childbirth in rural Bangladesh and the factors associated with physical IPV. The study also aims to investigate the association between IPV and maternal depressive symptoms after childbirth.

**Design:**

The study used cross-sectional data at 6–8 months postpartum. The sample included 660 mothers of newborn children. IPV was assessed by physical, emotional, and sexual violence. The Edinburgh Postnatal Depression Scale assessed maternal depressive symptoms.

**Results:**

Prevalence of physical IPV was 52%, sexual 65%, and emotional 84%. The husband's education (OR: 0.41, CI: 0.23–0.73), a poor relationship with the husband (OR: 2.64, CI: 1.07–6.54), and emotional violence by spouse (OR: 1.58, CI: 1.35–1.83) were significantly associated with physical IPV experienced by women. The perception of a fussy and difficult child (OR: 1.05, CI: 1.02–1.08), a poor relationship with the husband (OR: 4.95, CI: 2.55–9.62), and the experience of physical IPV (OR: 2.83, CI: 1.72–4.64) were found to be significant predictors of maternal depressive symptoms among women 6–8 months after childbirth. Neither forced sex nor emotional violence by an intimate partner was found to be significantly associated with maternal depressive symptoms 6–8 months postpartum.

**Conclusions:**

It is important to screen for both IPV and depressive symptoms during pregnancy and postpartum. Since IPV and spousal relationships are the most important predictors of maternal depressive symptoms in this study, couple-focused interventions at the community level are suggested.

Intimate partner violence (IPV) is defined by Cutcliffe and McKenna (1) as *physical, psychological or sexual mistreatment and/or other controlling behaviours such as economic or spiritual deprivation that are intended by the abuser to cause harm or are perceived by the victim to cause harm. It is a purposeful behaviour designed to achieve domination and control in the relationship* (1, p. 28).

*Prevalence of intimate partner violence:* A multi-country study by WHO reported that lifetime physical and/or sexual violence experienced by women varied between 15 and 71%, when compared with 4 and 54% experienced in the previous years (2). Prevalence data based on surveys conducted during 1998-2007 from 19 countries, in low- and high-income settings, report physical IPV experienced by women ranged between 2 and 64% (3). A lower and narrower range of prevalence is reported for IPV during pregnancy compared to other phases of the marital relationship. The frequency was 2% in Australia, Denmark, Cambodia, and the Philippines, while it was 13.5% in Uganda. The same study reported the prevalence of the experience of IPV the previous year was between 1% (Denmark) and 63% (DR Congo); IPV ever to range between 10% (Philippines) and 64% (DR Congo), and severe IPV ever between 5% (Azerbaijan) and 39.5% (Uganda) (3).

A study from Bangladesh reported almost no difference in the prevalence of IPV between rural and urban areas (4). Women reported an experience of IPV in the previous year to be 16% in the rural (19% in urban) area, IPV ever 42% in rural (40% in urban), and severe IPV ever 19% in both areas. A large study (*N*=8,320) from an urban area in Bangladesh reported that 55% of the husbands themselves claimed that they subjected their wives to physical IPV during their marriage, 23% in the previous year; 20% perpetrated sexual IPV; and 60% either physical or sexual IPV ever (5).

The majority (66%) of the women experiencing IPV both in urban and rural areas in Bangladesh do not disclose their experience of IPV nor do they seek help at all (4). When they do confide or seek help, it is to their parents (18%), siblings (16% in urban and 14% in rural), or neighbors (10% in urban and 12% in rural) that they turn. None reported seeking help from women's or non-governmental organizations (4).

*Impact of IPV on health*: Evidence indicates both direct and indirect adverse impacts of IPV during pregnancy. The direct impacts of IPV on women's reproductive health or pregnancy outcome include increased likelihood of miscarriage, premature labor or delivery, low birthweight of the newborn (6), higher levels of depression during and after pregnancy (7, 8), and insufficient weight gain during the pregnancy (8). Research also shows other adverse outcomes of IPV on women's physical and mental health, such as depression, anxiety, substance abuse (8), and injury and chronic pain (9). Indirect adverse impacts include infant and child mortality, particularly in the case of girls. This is reported to be higher among mothers exposed to IPV in India (10) and among educated mothers with exposure to IPV in Bangladesh (11). IPV exposure is reported to lead to a higher possibility of suicide ideation among women in Bangladesh (12, 13).

A recent systematic review and meta-analysis highlights the lack of evidence on the association between domestic violence and perinatal mental disorders (14). As pointed out in the review, most of the available evidence focuses on the impact of domestic violence on obstetric outcomes. This present study aims to describe the prevalence of IPV during pregnancy and 6–8 months after childbirth in rural Bangladesh, and the factors associated with physical IPV. Further, the study aims to investigate the association between IPV and maternal depressive symptoms after childbirth.

## Materials and method

This design of the study was cross-sectional and data were collected at 6–8 months postpartum from a larger longitudinal study titled ‘Risk factors and consequences of maternal perinatal depressive and anxiety symptoms: A community based study in the Bangladesh’ (15). The longitudinal study among rural Bangladeshi women was conducted during July 2008 to August 2009, following up women from the last trimester of pregnancy (baseline) until 6–8 months postpartum (second and final follow-up). The study was conducted in the rural parts of Mymensingh district, which has a population of about four million. It is predominantly agricultural and located 120 km north of Dhaka, the capital of Bangladesh. As in the rest of the country, approximately 40% of the population of Mymensingh lives under the national poverty line (16). The majority of the women were involved in unpaid domestic work including child care.

The longitudinal study, from which data of the current study are obtained, was approved by the Bangladesh Medical Research Council (BMRC/Eth.C/2008/402) in Bangladesh and the Regional Ethical Board at the Karolinska Institutet, Sweden (2008/919-31).

### Sample

At 6–8 months postpartum, 660 mothers with their infants remained of the original 720 women enrolled in the longitudinal study during their third trimester of pregnancy. Thus, 60 women, approximately 8%, were lost to follow-up during the period, due to maternal death at birth (*n*=2), neonatal and infant death (*n*=17), intrauterine death (*n*=1), stillbirth (*n*=25), multiple birth (*n*=3), and out-migration from the study area (*n*=12).

### Data collection

Data for the current study were collected using structured interviews. The interviews were conducted by trained interviewers at the respondents’ homes at 6–8 months after childbirth. The interviewers were university graduates and received 2-week-long training on data collection. Because the majority of the participants were illiterate or had little education, they were verbally informed before the interviews of the study objectives and of their right to refuse to participate or to terminate the interviews at any point.

*Socio-demographic data* included information on the respondent's and her spouse's age and completed years of schooling, parity (primi, multipara), number of children (1 child, 2–3 children, 4 or more children), per capita weekly household expenditure on food, and relationship with husband and mother-in-law after birth (good, in between/poor).

*IPV* was assessed by the instrument used by WHO in a multi-country study including Bangladesh (2). Physical violence by the husband after birth included six questions, scored yes or no: 1) slapped or thrown object at her, 2) pushed or shoved to the ground, 3) punched or hit, 4) kicked, dragged, or beat up, 5) burned on purpose, and 6) threatened to use weapon to hurt. No act of physical violence was scored 0 and acts of physical violence were scored 1–5. The women were asked about physical violence ‘ever’ (at any point in life), ‘during pregnancy’ and ‘since childbirth up to present time’ (6–8 months postpartum).

*Sexual violence* was assessed as forced sex by the husband since childbirth and up to present time (6–8 months postpartum) – that is, whether the woman reported that she was forced by her husband to have sexual intercourse (yes, no).

*Emotional violence* was assessed by seven items indicating controlling behavior of the husband – that is, whether the respondent's husband ever 1) tried to keep her from seeing friends, 2) restricted contact with her family, 3) insisted on knowing her whereabouts, 4) got angry due to jealousy, 5) was suspicious about her fidelity, and 6) expected to be asked permission before seeking health care for herself (2). No act of emotional violence was scored 0, and one or more acts of emotional violence were scored 1–6. The women were asked about emotional violence ‘ever’ and ‘since the childbirth until present time’ (6–8 months postpartum).

*Maternal depressive symptoms* were assessed by the Edinburgh Postnatal Depression Scale (EPDS) (17). The EPDS includes 10 items, scored on a 4-point scale (0–3). The scale rates the intensity of depressive symptoms within the last 7 days, and the higher the score, the more depressive symptoms. The items assess dysphoric mood (five items), anxiety (two items), guilt (one item), ability to cope with everyday life (one item), and suicidal thought (one item). The EPDS is used widely around the world. It also has been used in Bangladesh and validated by Gausia et al. (18), reporting sensitivity of the instrument to be 89%, specificity 89%, positive predictive value 40%, and negative predictive value 99%, using 9/10 as the cut-off score. This cut-off score was used in the current study to indicate presence of depressive symptoms as a discrete variable.

*The mother's perception of the infant's temperament* was assessed by the Infant Characteristic Questionnaire (ICQ) (19). The ICQ includes 24 items, comprising four sub-scales. All sub-scales are rated 1–7, a higher score indicating a more difficult temperament. The four sub-scales related to mother's perception of infant's temperament are 1) fussy and difficult (nine items), 2) unadaptable (five items), 3) unpredictable (six items), and 4) dull (four items). The ICQ has previously been used in Bangladesh (20).

### Data analyses

Descriptive analyses were performed to report sample descriptions and prevalence of IVP – physical, sexual, and emotional violence, and maternal depressive symptoms (EPDS). Univariate and multivariate logistics regressions were performed to calculate odds ratios (OR). The statistical significance of the OR was tested by confidence interval (CI) at 95%. Statistical Package for Social Scientists (SPSS, version 22) was used for all the data analyses.

## Results

The mean age of the women with newborn children was around 25 years, and their husbands’ almost 33 years (*p*<0.001; Table 1). The women in this sample were found to have significantly higher levels of schooling compared to their husbands (*p*<0.001). Twelve percent of the women reported having a poor relationship with their husbands and one-third (34%) with their mother-in-law.

**Table 1:** Background information of the women and their newborn children

	Women (*N*=660)	Husbands (*N*=660)
Age in years (Mean, SD)	24.7 (±6.1)	32.9 (±8.3)
Years of schooling (Mean, SD)	3.7 (±3.5)	3.2 (±4.0)
Poor relationship with husband	12%	
Poor relationship with mother-in-law (*n*=553)	34%	
Parity		
Primipara	28%	
2–3 children	53%	
4 or more children	19%	
Sex of the newborn child		
Girl	51%	
Boy	49%	
Perceived temperament of the newborn (*n*=657)		
Fussy and difficult (Mean, SD) [Min-Max]	25.9 (±7.5) [9.1–51.8]	
Unadaptable (Mean, SD) [Min–Max]	14.2 (±5.2) [4.2–29.4]	
Dull (Mean, SD) [Min–Max]	10.2 (±2.8) [3.2–19.7]	
Unpredictable (Mean, SD) [Min–Max]	14.5 (±3.9) [5.2–33.2]	
Maternal depressive symptoms 6–8 months after childbirth	32%	

The majority of the women in this study had two or more children, while 28% were first-time mothers. Sex distribution of the newborn children was almost equal. Almost one third of the women (32%) indicated maternal depressive symptoms, according to the EPDS, 6–8 months after childbirth (Table 1).

### Prevalence of IPV

As shown in Table 2, 70% of the women reported having suffered physical violence from their spouses during their marriage. Almost one-fifth (18%) were subjected to physical violence by their partner during pregnancy, and more than half of the women (52%) reported that their husbands were physically violent with them 6–8 months after childbirth. Sixty-five percent of the women reported being forced to have sex against their will 6–8 months after childbirth.

**Table 2:** Intimate partner violence experienced by women 6–8 months after childbirth (*N*=660)

Components of intimate partner violence	Percent
Physical violence (ever)	70
Physical violence during pregnancy	18
Physical violence 6–8 months after childbirth	52
Sexual violence	65
Emotional violence	84

Eighty-four percent of the women reported emotional violence by their intimate partner, the most common issue being expected to ask the partner's permission for seeking health care for herself (72%) and the partner's anger based on jealousy (55%) (Table 2).

### Predictors of physical IPV

Women whose husbands had five or more years of schooling were less likely (OR: 0.41, CI: 0.23–0.73) than illiterate and less-educated husbands to experience physical IPV (Table 3). Women who reported poor relationships with their husbands were more likely (OR: 2.64, CI: 1.07–6.54) than those with good relationships with their husbands to experience physical IPV. The more emotional violence the women were subjected to, the greater was the likelihood (OR: 1.58, CI: 1.35–1.83) that they would also be subjected to physical violence by an intimate partner.

**Table 3:** Predictors of physical intimate partner violence experienced by women 6–8 months after childbirth (*N*=660)

	Odds ratio (95% CI)
Parity§	
Primipara	1.00
2–3 children	1.79 (1.00–3.19)
4 or more children	1.95 (0.89–4.29)
Husband's education§	
Illiterate	1.00
1–4 years of schooling	0.80 (0.49–1.32)
Five or more years of schooling	0.41 (0.23–0.73)
Relationship with husband§	
Good	1.00
Poor	2.64 (1.07–6.54)
Relationship with mother-in-law§	
Good	1.00
Poor	2.03 (1.31–3.16)
Emotional violence§	1.58 (1.35–1.83)

§ Adjusted for woman's and husband's age and education, per capita daily household expenditure, child's temperament, and the other covariates mentioned in the table.

### Predictors of maternal depressive symptoms 6–8 months after childbirth

As indicated in Table 4, those having a newborn child whose temperament was perceived by them to be fussy and difficult, as assessed by the ICQ, were more likely to report depressive symptoms 6–8 months after childbirth (OR: 1.05, CI: 1.02–1.08). Women reporting poor relationships with their husbands were five times more likely (OR: 4.95, CI: 2.55–9.62) to indicate maternal depressive symptoms than those with good spousal relationships. Women subjected to physical IPV after childbirth (OR: 2.83, CI: 1.72–4.64) were almost three times more likely than those not reporting IPV to show maternal depressive symptoms 6–8 months after childbirth. Neither sexual nor emotional violence by an intimate partner was found to be significantly associated with maternal depressive symptoms 6–8 months after childbirth.

**Table 4:** Predictors of maternal depressive symptoms 6–8 months after childbirth (*N*=660)

	Odds ratio* (95% CI)
Fussy and difficult child	1.05 (1.02–1.08)
Poor relationship with husband	4.95 (2.55–9.62)
Physical intimate partner violence after childbirth	2.83 (1.72–4.64)
Sexual violence	1.09 (0.73–1.64)
Emotional violence	1.05 (0.90–1.22)

* Adjusted for woman's and husband's age and education, per capita daily household expenditure, child temperament, relationship with mother-in-law, controlling behavior of husband and the covariates mentioned in the model.

### Association of economic status and sex of the newborn with physical IPV and maternal depressive symptoms

Table 5 shows that household economic status and the sex of the newborn had no statistically significant association with either physical IPV or maternal depressive symptoms 6–8 months after childbirth. In the case of emotional violence, no statistically significant association was found in the bivariate analysis with either household economic status or sex of the newborn (data not shown). Sexual violence indicated no such association either with household economic status. However, the bivariate analysis indicated a statistically significant association between sexual violence and sex of the newborn, but this significance disappeared when other co-variates were entered in the mode (Table 5).

**Table 5:** Association of economic status and sex of the newborn child with physical and sexual intimate partner violence (IPV), and maternal depressive symptoms 6–8 months after childbirth (MD) (*N*=660)

	Sexual violence odds ratio (95% CI)	IPV odds ratio (95% CI)	MD odds ratio (95% CI)
Sex of the newborn*			
Girl	1.00	1.00	1.00
Boy	1.16 (0.82–1.66)	0.75 (0.55–1.03)	0.84 (0.60–1.16)
Per capita daily household expenditure§		1.00 (0.99–1.02)	0.99 (0.97–1.01)

* Univariate analysis;

§ adjusted for woman's and husband's age and education, parity, child's temperament, relationship with of woman with husband and mother-in-law and emotional violence by husband.

## Discussion

The main results in this study show a high prevalence of IPV at 6–8 months postpartum among rural Bangladeshi women. IPV was significantly associated with a low educational level of the husband, a poor relationship with the spouse and the mother-in-law, and emotional violence – in other words, the spouse's controlling behavior over the woman. The mother's perception of the infant as fussy and difficult, a poor relationship with the husband, and physical IPV were found to be significant predictors of maternal depressive symptoms among women 6–8 months after childbirth. Physical IPV, but not sexual or emotional violence, was associated with a greater likelihood of reporting maternal depressive symptoms 6–8 months after childbirth. It is important to note that these results are applicable for married women in rural Bangladesh, as the study was conducted with married women only.

The high prevalence of IPV experienced by women in this study is consistent with that reported in other studies from Bangladesh (2, 4, 5). Garcia-Moreno and colleagues (2) reported a prevalence of physical and sexual violence ever of 62% in a Bangladesh rural area. In the current study, 52% of the women reported physical IPV and 65% that they been forced to have sexual intercourse 6–8 months postpartum. Azziz-Baumgartner and colleagues (21), in a recently published study on low-income Bangladeshi women and displaced ethnic Biharis in Bangladesh, reported the prevalence of physical IPV to be 58% after childbirth and IPV ever during the relationship at 80%. Almost every fifth woman (18%) in our study reported physical violence during the pregnancy. This is higher than reported in a multi-country study, including 19 high- and low-income countries, which report the prevalence of IPV during pregnancy to range between 2 and 13.5% (3).

Consistent with findings from Bangladesh (22) and the United Kingdom (23), socio-economic status was not associated with IPV in the current study. However, the husband's low education emerged as a predictor for IPV experienced by women 6–8 months after childbirth. An inverse association is reported between educational level, in both women and men, and IPV in general (2) and after birth (21). In addition, either the woman or her husband achieving secondary education was associated with decreased IPV in a multicountry study (24). In our study, we found that the husband's education of 5 years or longer was protective of IPV experienced by women. A similar finding was reported by Naved and Persson (22) in rural Bangladesh. In the current study, the mothers were more educated than their spouses. Inequality in educational level between partners is suggested to increase the risk of women experiencing IPV (24).

This study clearly indicates that physical IPV after childbirth leads to a greater likelihood of postpartum maternal depressive symptoms. This is consistent with findings of a study by Valentine and colleagues (25) that reports recent pre-natal IPV to be a strong predictor of postpartum depression among Latinas in Los Angeles. In a systematic review and meta-analysis of 32 cross-sectional and five cohort studies, Beydoun and colleagues (26) summarize that the exposure to IPV increased the risk of both major depression and postpartum depression among women. Ludermir and colleagues (7) showed an increased risk for postpartum depression if the mother reported IPV during pregnancy. The highest risk was in women reporting all kinds of IPV (physical, sexual, and psychological violence), but psychological violence was more strongly related to postpartum depression than physical and sexual violence.

In our study, we found that only physical violence and not emotional violence by the spouse predicted maternal depressive symptoms at 6–8 months. The majority of Bangladeshi men feel that a wife is accountable to her husband for her behavior and that violence is an acceptable form of corrective punishment (27). Consistent with other research (28, 29), this study showed that a poor spousal relationship is an important vulnerability factor for women, that has a significant impact on reporting postpartum depressive symptoms. Astbury (30) explained that in a patriarchal society like Bangladesh, where men control the family and wealth, and women are legally restricted in seeking divorce, the rural women do not necessarily recognize a slap or a shove as violence. Qualitative data collected in the larger study from which the current study is derived indicate that the women felt that their husbands had the right to slap or shove them in case of ‘minor offences’ (data unpublished). These were not recognized as any form of violence. Although 12% of the women in this study acknowledge a poor relationship with their husbands, its impact on the mental health of the women is clearly noticeable in their increased likelihood of reporting depressive symptoms. The association between partner relationship and postpartum depressive symptoms reported in other research is inconclusive. In a meta-analysis, Beck (31) reported poor marital relationship to significantly predict postpartum depression. On the contrary, a systematic review conducted by Lancaster et al. (32) reported that the 11 studies that included information on association between quality of partner relationship and depressive symptoms during pregnancy, found no significant association between the two variables.

Research indicates that women who experienced IPV during pregnancy were likely to perceive their infants to be temperamentally irritable and difficult (33, 34). Our findings suggest that the mother's perception of an infant as fussy and difficult increases the likelihood of reporting depressive symptoms. It is beyond the scope of the study to discuss the relationship between IPV and perceived child temperament and how it impacts upon maternal depressive symptoms.

Finally, although we found a lower prevalence of IPV during pregnancy than postpartum in our study, research has shown that IPV during pregnancy could be a gateway for further violence (7). Several valid and reliable instruments can be found to screen for IPV (35, 36), but their effective implementation can be challenging (37). As indicated by a Canadian study (38), it is important to screen for violence among women in relation to mental health consequences and not only for physical symptoms. As indicated by the results of our study, it is important to screen for both IPV and depressive symptoms during pregnancy and postpartum.

Given that IPV and spousal relationship are the most important predictors of maternal depressive symptoms in this study, couple-focused interventions at the community level are suggested to reduce maternal depressive symptoms. In the Bangladesh context, community health workers can screen for IPV and depressive symptoms during antenatal check-ups of pregnant women and the first postpartum period. Given the lack of formal health facilities in rural Bangladesh, community health workers can be trained to provide initial counseling to those detected with depressive symptoms and refer to nearby hospitals if need be.
